# Letter to the Editor on “Leveraging Biomedical Engineering Engineers to Improve Obstructive Sleep Apnea (OSA) Care for Our Stroke Patients”

**DOI:** 10.1109/JTEHM.2023.3318930

**Published:** 2023-09-29

**Authors:** Sara E. Benjamin, Charlene E. Gamaldo

**Affiliations:** Neurology DepartmentThe Johns Hopkins School of Medicine 1500 Baltimore MD 21287 USA

## Abstract

Obstructive sleep apnea (OSA), a condition of recurring, episodic complete or upper airway collapse, is a common disorder, affecting an estimated 17.4% of women and 33.9% of men in the United States [1]. The first line treatment for OSA is Continuous Positive Airway Pressure (CPAP) therapy, a medical device that delivers adequate airflow and oxygenation during sleep by way of a tube that connects an air compressor to a face mask that can fit over the nose, under the nose, or over the nose and mouth.

Obstructive sleep apnea (OSA), a condition of recurring, episodic complete or upper airway collapse, is a common disorder, affecting an estimated 17.4% of women and 33.9% of men in the United States [1]. The first line treatment for OSA is Continuous Positive Airway Pressure (CPAP) therapy, a medical device that delivers adequate airflow and oxygenation during sleep by way of a tube that connects an air compressor to a face mask that can fit over the nose, under the nose, or over the nose and mouth.

OSA is recognized as a common comorbid condition with other medical and neurological conditions including stroke, a prevalent condition, suffered by an estimated 800,000 people annually, and the most common cause of new disability. Seventy percent of all post-stroke patients have un/undertreated mild sleep apnea and 40% moderate—severe apnea [2]. The risk of recurrence of stroke at five years is now about 12% and has stayed there for the past twenty years [3]. The Centers for Disease Control (CDC) reports that stroke-related costs (related to medications, lost work, and health care services utilization) between 2018 and 2019 were estimated at U.S. ${\$}56.5$ billion [4].

The American Heart Association now lists sleep apnea as both a primary and secondary risk factor for stroke [5]. In addition to the potential elevated risk of stroke recurrence, untreated apnea has been associated with reduced outcomes and recovery. Inversely, common stroke-related motor deficits such as hemiplegia can serve as a barrier to OSA treatment adherence due to the challenges of putting the CPAP mask on or off independently. This can result in sleep disruption and a health burden risk to the caregiver who will need to assist the patient with the nightly CPAP (re)application. Mask designers, occupational therapists, and engineers should work together to develop CPAP masks that can be independently applied across a range of dexterity capabilities. For instance, developing a mask that can be placed securely with a single hand by hemiparetic post-stroke patients that also incorporates technology to confirm proper mask position and airflow along with troubleshooting capabilities for improperly placed masks. Trials should be done in stroke patient populations specifically to test the feasibility of using these types of home medical devices in the context of potential motor and/or cognitive limitations.

Other neurological conditions often associated with sleep disorders could also benefit from biomedical engineering strategies to improve OSA treatment and overall outcomes. For example, patients with dementia experience high rates of insomnia, sleep-disordered breathing, and daytime sleepiness [6] and patients with Parkinson’s disease (over 80% of patients with Parkinson’s disease are thought to have some degree of sleep disturbance) [7] could also benefit from biomedical engineering strategies to improve OSA treatment and overall outcomes.

This work with durable medical equipment is only one of many avenues through which biomedical engineers could benefit sleep patients. For example, recent work has looked at non-REM sleep with hypertonia in frontopolar EEG signal analysis in neurodegenerative sleep-related conditions, for example, differentiating Parkinsonian syndromes from other forms of dementia [8]. This highlights the importance of more cross-pollination between biomedical engineers and clinical and research sleep care teams to help advance the health outcomes of sleep patients.

To meet the clinical needs of patients with neurological conditions, biomedical engineers should spend time getting to know the unique challenges associated with living with these conditions. For example, a biomedical apprenticeship could include observation in clinical spaces with rotations in the neurology clinic, primary care clinic, and home healthcare teams, and with physical, occupational, and speech therapists to appreciate the unique challenges of these patient populations. With the increased reliance on technology for sleep diagnosis and treatment, sleep fellow training programs should offer exposure to biomedical engineering experts and researchers who can enlighten the trainees on the possibilities these technologies hold. Patients and caregivers should also be engaged to provide ongoing insight into care gaps and unmet needs that could be filled with engineering advances.
